# Supplementary material for: The interactome of intact mitochondria by cross-linking mass spectrometry provides evidence for coexisting respiratory supercomplexes
Source: Mol Cell Proteomics. 2017 Dec 8;17(2):216–32. doi: 10.1074/mcp.RA117.000470 (PMC5795388; doi:10.1074/mcp.RA117.000470)
Supplement: Supplemental Data [file supp_RA117.000470_133922_0_supp_23978_fzffwf.zip › spectra_annotation/mito_spectra_annotation/388-1-4-1-4-1.pdf]

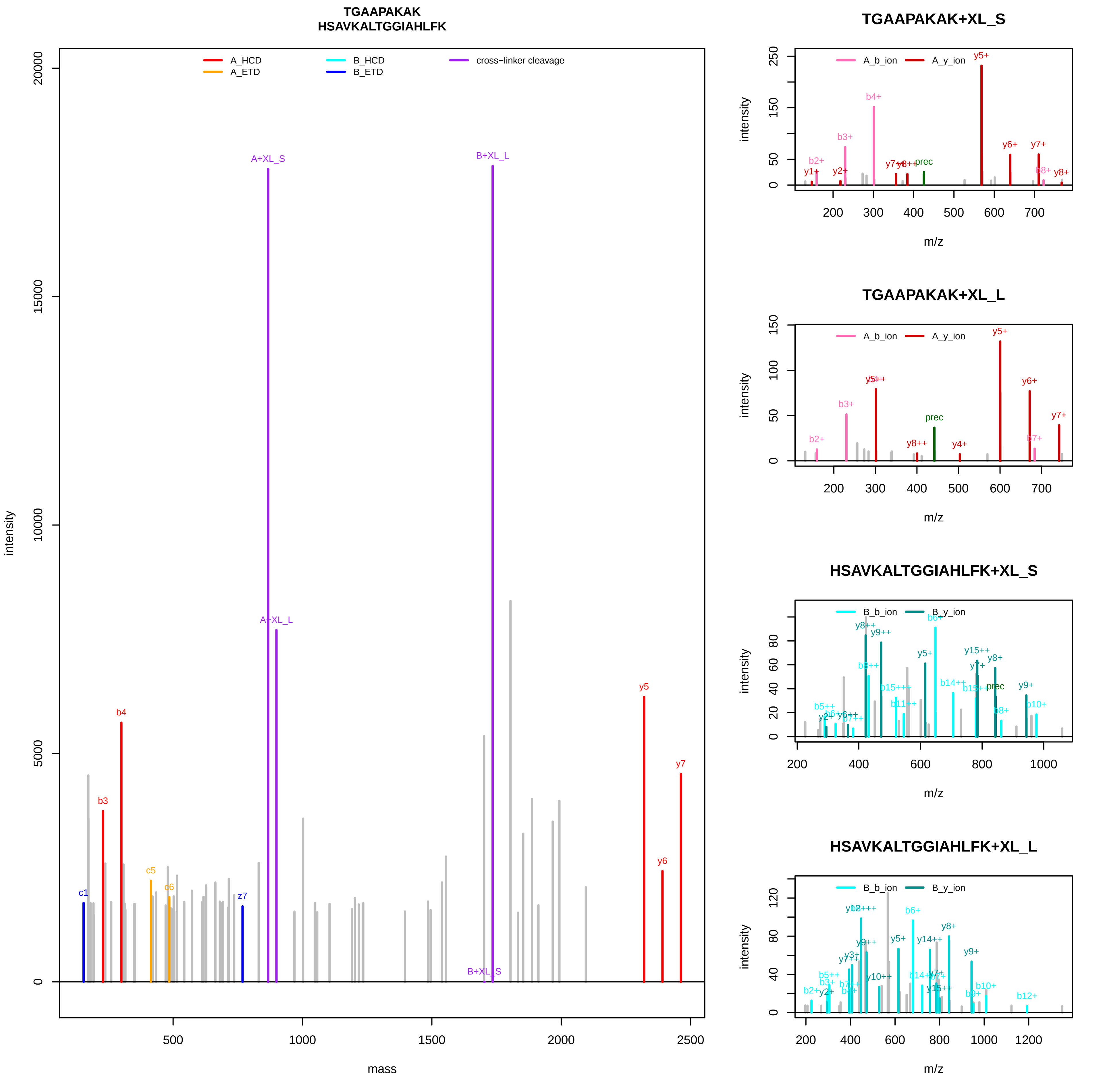
TGAAPAKAK
HSAVKALTGGIAHLFK
A_HCD
A_ETD
B_HCD
B_ETD
cross−linker cleavage
A+XL_S
B+XL_L
A+XL_L
B+XL_S
b3
b4
c1
c5
c6
z7
y5
y6
y7
intensity
mass
0
5000
10000
15000
20000
500
1000
1500
2000
2500
TGAAPAKAK+XL_S
A_b_ion
A_y_ion
y5+
b4+
b3+
b2+
y1+
y2+
y6+
y7+
y8+
prec
TGAAPAKAK+XL_L
y5++
y8++
y4+
b7+
HSAVKALTGGIAHLFK+XL_S
B_b_ion
B_y_ion
y8++
y9++
y5+
y15++
y8+
y9+
b10+
b5++
y2+
y6++
b14++
HSAVKALTGGIAHLFK+XL_L
b6+
y8+
y5+
y14++
y9+
y10++
b10+
b12+
b2+
y15++
m/z
